# Underwater Processing of Materials

**DOI:** 10.3390/ma15144902

**Published:** 2022-07-14

**Authors:** Dariusz Fydrych, Jacek Tomków

**Affiliations:** Institute of Manufacturing and Materials Technology, Faculty of Mechanical Engineering and Ship Technology, Gdańsk University of Technology, Gabriela Narutowicza Street 11/12, 80-233 Gdańsk, Poland

Technological processes carried out in the water environment include the production and processing of engineering materials and giving them specific properties [1,2]. A large number of complex processes must be carried out underwater out of necessity, for example, when there is no technical possibility to lift the structural elements above the water surface, or this is economically unjustified [3,4]. In these cases, the water environment can generate serious technological and metallurgical problems (structural changes, cracking, porosity, stress, deformation, etc.) [5,6,7]. Processes that might be used in these circumstances include, for example, welding (arc, laser, adhesive bonding), cutting and coating, surfacing, and thermal spraying [8]. On the other hand, the physicochemical properties of water can be attractive and used efficiently, e.g., to promote the specific properties of certain materials. Typical processes that use water in this way are, e.g., friction stir welding, plasma cutting, and laser remelting [9]. The practical application of underwater technologies requires the development of techniques for testing the quality of processed materials and advanced automated and robotic applications, and their ability to operate in difficult conditions, e.g., at great depths or in conditions harmful to health [10,11].

Since the demand for materials and technologies, including the design and control of mechanized and robotic systems used in the water environment, is still increasing exponentially, experimental and simulation studies are an important factor contributing to their wider use [12].

The purpose of this Special Issue is to present the latest developments in the field for processing materials in a water environment, especially at offshore and nuclear plant structures. This includes technologies related to manufacturing, properties, degradation, failures, protection, maintenance and repairs, joining, and the cutting of materials. This Special Issue mainly focuses on assessing the influence of the environment and technology on the behavior of materials underwater. Eleven research articles are published as part of this Special Issue, six of which are devoted to underwater welding.

In article [13], a waterproof coating application for the surface of covered electrodes is investigated. It is proven that paraffin wax as an additional substance allows reduced hardness in the HAZ of pad welds by 40–50 HV10. Moreover, it reduces the diffusible hydrogen content in deposited metal by 35% in comparison with electrodes without waterproof coating and by 24% in comparison with electrodes intended for underwater welding. A continuation of works aiming to determine the effectiveness of waterproof coatings on the weldability of steel underwater is presented in the article [14]. This work confirms the results observed in previous investigations. It is stated that the usage of paraffin wax as a protective coating on the surface of the filler material improves the weldability of the investigated material. The HAZ hardness values decrease by 30–40 HV10, and at many measurement points such values are lower than 380 HV10, which is the required level for S460N steel air-welded joints. Moreover, this reduced the susceptibility of steel to cold cracking.

The content of article [15] shows that the number of imperfections has a higher influence on the tensile strength of GMAW underwater-welded joints of S460N steel than the welding environment. From the tensile strength tests carried out, it can be concluded that joints with a certain number of welding imperfections can transfer tensile stresses at the level of the base material. In a complex state of stress, such as bending, torsional, or tensile stress, the welding imperfections (e.g., pores) significantly decrease the welded joint properties.

In article [16], research is undertaken to verify the possibility of applying the recommendations of the DIN EN ISO 3690 standard in wet welding conditions. The records of the standard are analyzed for: sample dimensions and the number of samples taken simultaneously; time limitations defined by the standard regarding the welding and the cleaning process; the time, temperature, and method defined for the analysis of the diffusible hydrogen content; and the normalization of the hydrogen concentration measured. Moreover, article [17] presents the results of investigations aimed at reducing the susceptibility of steel welded underwater to cold cracking through the use of induction heating. It is found that preheating reduces the diffusible hydrogen content by up to 30%, but does not significantly reduce the hardness of the joints.

Other issues that are very important from a practical point of view are described in article [18]. The results of research on the influence of underwater wet welding polarity and simulated welding depth on the behavior of the rutile electrodes, arc stability, welding efficiency, and weld bead geometry are presented. In particular, the authors propose interesting models illustrating the electric arc phenomena and the resulting penetration of the wet welds depending on the polarity of the welding.

Another technological process carried out in the water environment is the cutting of structural elements. Article [19] is devoted to the issues of underwater cutting with the use of flux-cored wires. The main achievement of the authors is the development of a multi-phase mechanism of underwater wet flux-cored cutting consisting of working and idle cycles of the electrical process in a pulsating vapor gas bubble.

The issues of exploitation and the use of natural energy sources, currently often described in the scientific literature, also apply to the processes and materials used in the air environment [20,21,22]. The processes used to manufacture welded joints are significant both offshore and in the energy industry. Several articles on this subject are published in the Special Issue.

The results of research on the plasticity of bead-on-plate welds made using two types of seamless, copper-plated, flux-cored wires stored in two locations are presented in article [23]. The test results show that changes in storage conditions strongly affect the quality of welding wires and the mechanical properties of the welded joints made with them.

The authors of [24] verify the possibility of using the electromechanical impedance method to detect defects in welded joints made of steel intended for the offshore industry. The proposed NDT method is proven effective in detecting sub-millimeter cracks in steel-welded joints.

Articles [25,26] are devoted to the issues surrounding the weldability of dissimilar steels joints for the energy industry. The paper describes the behavior of dissimilar materials subjected to laser welding, for example, P91 steel and INCOLOY 800HT nickel alloy. It is stated that the solidification cracking phenomenon occurs in the weld zone only due to high heat input from the laser welding and invariable cooling due to the presence of dissimilar alloys, and that the tensile strength and impact toughness of the investigated joints meet the boiler requirement [25].

In the second article of this research team, the weldability of elements from P91 and P22 steels during welding with the GTAW process is determined. The authors conclude that the inhomogeneity of mechanical properties (impact strength and hardness) across the as-welded joint is reduced after post-weld heat treatment [26].

These Special Issue articles have been cited 122 times so far. This proves the topicality and importance of the subjects discussed within them. The continuation of the subject of this Special Issue is a Topic entitled “Welding and Joining of Materials in Off-shore and Energy Industry”, which intends to collate articles published in the following journals: *Materials, Metals, Coatings, Journal of Manufacturing and Materials Processing*, and *Applied Sciences* [27,28,29,30]. The international Topic Editorial Board watches over the quality of the articles, as does the MDPI Editorial Office. We would like to thank the authors for submitting original research articles related to the underwater processing of materials. Moreover, we would also like to thank the reviewers of the articles and the academic editors for their commitment. We extend separate expressions of gratitude to Ms. Agnes Zhou for her invaluable support to the editors, and all MDPI staff involved in the publishing process that has contributed to the success of our Special Issue.
